# Author Correction: Layered subsurface in Utopia Basin of Mars revealed by Zhurong rover radar

**DOI:** 10.1038/s41586-023-05734-0

**Published:** 2023-01-24

**Authors:** Chao Li, Yikang Zheng, Xin Wang, Jinhai Zhang, Yibo Wang, Ling Chen, Lei Zhang, Pan Zhao, Yike Liu, Wenmin Lv, Yang Liu, Xu Zhao, Jinlai Hao, Weijia Sun, Xiaofeng Liu, Bojun Jia, Juan Li, Haiqiang Lan, Wenzhe Fa, Yongxin Pan, Fuyuan Wu

**Affiliations:** 1 Key Laboratory of Earth and Planetary Physics, Institute of Geology and Geophysics, Chinese Academy of Sciences, Beijing, China; 2 Key Laboratory of Petroleum Resource Research, Institute of Geology and Geophysics, Chinese Academy of Sciences, Beijing, China; 3 College of Earth and Planetary Sciences, University of Chinese Academy of Sciences, Beijing, China; 4 State Key Laboratory of Lithospheric Evolution, Institute of Geology and Geophysics, Chinese Academy of Sciences, Beijing, China; 5 State Key Laboratory of Space Weather, National Space Science Center, Chinese Academy of Sciences, Beijing, China; 6 Center for Excellence in Comparative Planetology, Chinese Academy of Sciences, Hefei, China; 7 Institute of Remote Sensing and Geographical Information System, School of Earth and Space Sciences, Peking University, Beijing, China

**Keywords:** Seismology, Structural geology, Sedimentology

Correction to: *Nature* 10.1038/s41586-022-05147-5 Published online 26 September 2022

In the version of this article initially published, in Fig. 1a, the longitude of the Viking-2 landing site was wrongly shown at 225.7E, and has now been corrected to appear at 225.7W in the HTML and PDF versions of the article.

